# Modulating Effects of Contextual Emotions on the Neural Plasticity Induced by Word Learning

**DOI:** 10.3389/fnhum.2018.00464

**Published:** 2018-11-23

**Authors:** Jingjing Guo, Dingding Li, Yanling Bi, Chunhui Chen

**Affiliations:** ^1^Key Laboratory of Behavior and Cognitive Neuroscience in Shaanxi Province, School of Psychology, Shaanxi Normal University, Xi'an, China; ^2^State Key Laboratory of Cognitive Neuroscience and Learning, Beijing Normal University, Beijing, China

**Keywords:** contextual emotions, fMRI, word learning, concreteness, brain plasticity

## Abstract

Recently, numerous studies have investigated the neurocognitive mechanism of learning words in isolation or in semantic contexts. However, emotion as an important influencing factor on novel word learning has not been fully considered in the previous studies. In addition, the effects of emotion on word learning and the underlying neural mechanism have not been systematically investigated. Sixteen participants were trained to learn novel concrete or abstract words under negative, neutral, and positive contextual emotions over 3 days; then, fMRI scanning was done during the testing sessions on day 1 and day 3. We compared the brain activations in day 1 and day 3 to investigate the role of contextual emotions in learning different types of words and the corresponding neural plasticity changes. Behaviorally, the performance of the words learned in the negative context was lower than those in the neutral and positive contexts, which indicated that contextual emotions had a significant impact on novel word learning. Correspondingly, the functional plasticity changes of the right angular gyrus (AG), bilateral insula, and anterior cingulate cortex (ACC) induced by word learning were modulated by the contextual emotions. The insula also was sensitive to the concreteness of the learned words. More importantly, the functional plasticity changes of the left inferior frontal gyrus (left IFG) and left fusiform gyrus (left FG) were interactively influenced by the contextual emotions and concreteness, suggesting that the contextual emotional information had a discriminable effect on different types of words in the neural mechanism level. These results demonstrate that emotional information in contexts is inevitably involved in word learning. The role of contextual emotions in brain plasticity for learning is discussed.

## Introduction

Word learning is a critical way for individuals to improve their language ability and acquire scientific and cultural knowledge. It is of great importance to understand the role of brain plasticity in mastering the vocabulary rules as it may enhance the process of language learning. A number of researchers have examined the neurocognitive mechanism of learning words in isolation or in semantic contexts. However, as an important influencing factor of novel word learning, emotion has not been fully considered in the previous studies. Moreover, the effect of emotion on word learning and its underlying neural mechanism have not been systematically investigated. Therefore, this study aimed to explore the mechanisms of word learning in emotional contexts.

Large number of research have been investigating the neural mechanisms of isolated word learning using offline or online learning paradigm (Breitenstein et al., 2005; Xue et al., 2006, 2010; Raboyeau et al., 2010; Song et al., 2010; Minicucci et al., 2013; Zhao et al., 2014). Results showed that word learning experience could induce functional plasticity changes of the inferior frontal gyrus (IFG), inferior parietal lobule [angular gyrus (AG)/supramarginal gyrus], occipital-temporal cortex, temporal-parietal cortex, and other brain regions, and the activation in the hippocampus played an important role in lexical acquisition and could predict the learning performance. However, in the real situations of word learning, people mainly obtain or construct the meaning of words through the corresponding contextual information rather than passively receive the connections between word form and meanings. Recently, researchers began to focus on the mechanism of word learning in semantic contexts (Mestres-Missé et al., 2009; Chen et al., 2014; Dautriche and Chemla, 2014). Mestres-Missé et al. (2009) used a real-time online learning task to explore the neural mechanisms of developing the mapping between a novel word and the lexical meaning in the semantic context. They offered a high or low restricted sentential context to participants and asked them to guess the meaning of the target novel word. For instance, they firstly presented “after the meals you should brush your” and then the target novel word “tankies,” and the participants were expected to get the meaning of “tankies,” teeth. Through a series of studies, they found that the semantic mapping process was involved with the IFG, middle temporal gyrus (MTG), parahippocampal gyrus, and some sub-cortex regions, and the different types of word learning (abstract vs. concrete words; verb vs. noun) elicited different patterns of neural network activity (Mestres-Missé et al., 2008, 2009, 2010). Moreover, a further functional connectivity analysis found that the left IFG network seemed to be involved in mapping the selected meaning among the competing candidates onto the new words, and the left MTG network might be implicated in integrating the newly learned words into the corresponding sentential contexts (Ye et al., 2011).

Previous research on word learning was mainly based on the concept of “pure cognition,” whereas the role of emotion in word learning was seldom considered. But in real life, language learning and emotions are inseparable. In recent years, with the rapid development of affective neuroscience, researchers began to pay attention to the influence of emotions on cognitive processing (Pessoa, 2008; Schmitz et al., 2009; Pessoa and Adolphs, 2011; Righi et al., 2012; Pourtois et al., 2013; Dolcos and Denkova, 2014). Emotions were frequently reported to be very important for the processing of words (Schacht and Sommer, 2009; Sutton and Altarriba, 2011). On the one hand, an individual's emotional state played an important role in word processing (Gray et al., 2002; Guo et al., 2018). Fitzgerald et al. (2011) examined how happy and sad moods impacted memory for valenced stimuli (positive, negative, and neutral words). Their findings indicated that the orbitofrontal cortex was implicated in successful recall for mood-congruent negative words, while the left IFG and middle frontal gyrus (MFG) showed enhanced activation for mood-incongruent words. In addition, a brain imaging study found that depressed patients showed weakened activations in the frontotemporal and limbic regions for positive words but an enhanced activation in the inferior parietal lobule for negative words (Canli et al., 2004). On the other hand, the emotional information contained in a word had an important influence on the processing of the word itself and the adjacent stimuli (Nakic et al., 2006; Guo et al., 2011; Kazanas and Altarriba, 2016). Sutton and Altarriba (2011) adopted a modified version of the attentional dot probe paradigm and found that participants responded faster to the probe when it appeared in the same location as the negative words compared with the neutral words, which suggested that the words' emotional information was accessed automatically, influencing the attentional catch for the probes.

Consistent with the ecological tendency in psychological research, some researchers also have begun to pay attention to the influence of contextual emotional information on word learning (Smith et al., 2004; Medford et al., 2005; Beaucousin et al., 2007). For example, a recent study asked participants to remember words which were superimposed centrally onto positive, negative, or neutral picture contexts during learning and to fulfill an “old/new” judgment for learned or new words during testing (Zhang et al., 2015). The results revealed that negative and high-arousing positive contexts impaired the recognition of words compared with the neutral context, which suggested that the influence of contextual emotion on word learning cannot be ignored. Smith et al. (2004) found that emotionally neutral pictures presented on an emotional background context compared with neutral context during the encoding phase enhanced the activations in areas implicated in episodic memory, such as parahippocampal cortex, in the subsequent memory test. Moreover, Medford et al. (2005) revealed that encoding aversive sentences caused additional significant activations over matched neutral sentences in the left anterior cingulate gyrus and left precuneus. All the research mentioned above agreed that contextual emotions had an important influence on word processing or comprehension. However, when it comes to novel word learning, which requires an individual to form new mapping between words' grapheme and conceptual meaning, there is substantially rare evidence. Therefore, this study explores the modulating effects of contextual emotions on neural plasticity involved in novel word learning by combining offline behavioral training and fMRI scanning.

Notably, word concreteness, which is an intrinsic property of a word, played a significant role in word learning besides the external factors (such as learner's state or contextual information) (Mestres-Missé et al., 2008, 2009). Mestres-Missé et al. (2009) found that concrete words elicited greater activation than abstract words in the ventral anterior part of the fusiform gyrus (FG), which is associated with higher levels of visual processing. More importantly, researchers began to realize that emotionality and concreteness of words had close interactions with each other (Vigliocco et al., 2014). Altarriba et al. (1999) firstly proposed that the emotional information of words might affect the concreteness of the words. Kousta et al. (2011) revealed that abstract words were more emotionally valenced than concrete words, which accounted for a residual latency advantage for abstract words, when variables (such as imageability and context availability) were held constant. A recent study that used a lexical judgment task revealed that the processing of emotional words was modulated by word concreteness (Yao and Wang, 2013). Thus, this study also considered the concreteness of the target words as a variable that interacts with contextual emotions.

To investigate the brain functional plasticity changes induced by word learning, we conducted the offline behavioral training and fMRI testing during 3 continuous days and compared the differences in brain activations for different learning contexts. Based on the results of previous studies, we assumed that (1) contextual emotions have significant impact on a brain region's functional plasticity induced by word learning, specifically, the activation of word learning related areas, such as left IFG, left MTG, right AG, FG, amygdala, etc., would be modulated by the contextual emotions; and (2) the concreteness and the emotional context of the target word might also have interactive effects on word learning. Specifically, plasticity changes induced by learning of concrete words would be significantly modulated by contextual emotions but the learning of abstract words would not. Because abstract words were found to be more emotionally valenced than concrete words, emotionality was more insensitive to the environmental emotional information like contextual information.

## Experiment

### Methods

#### Participants

Sixteen native Chinese speakers, eight males and eight females, aged 18–24 years (*M* = 20.7, *SD* = 1.1), participated in the experiment and were paid after fulfilling all the experimental sessions. All were screened using checking lists issued by the Brain Imaging Centre of National Key Laboratory of Cognitive Neuroscience and Learning in Beijing Normal University to make sure that they had no neurological or psychiatric disorders. They were all right-handed with normal vision or corrected to normal vision, and their recent emotional state was stable. All participants signed informed consents approved by the Institutional Review Board of Beijing Normal University.

#### Design

3 (Contextual emotions: positive, negative, and neutral) ^*^ 2 (Concreteness of words: abstract, concrete) ^*^ 2 (Training days: day 1, day 3) within-subject design was adopted.

#### Materials

Firstly, 360 real words were selected from the Chinese Affective Word System (Wang et al., 2008) with equal number of positive, negative, and neutral words. Twenty additional participants who did not take part in the formal experiment rated the emotional valence, arousal, familiarity, and concreteness of these words on a 9-point Likert scale where 1 and 9 represented minimum and maximum degrees, respectively. According to the evaluation results, we chose 180 Chinese two-character nouns; each learning condition (three emotions by concreteness) had 30 items. The corresponding information for each condition is shown in Table 1. Statistical analyses indicated that the effects of valence, arousal, and concreteness reached significance [*F*_(2, 118)_ = 879.15, *p* < 0.001, η^2^ = 0.82; *F*_(2, 118)_ = 175.4, *p* < 0.001, η^2^ = 0.64; *F*_(1, 89)_ = 3001.4, *p* < 0.001, η^2^ = 0.93, respectively]. *Post-hoc* comparisons revealed that the valence of positive, negative, and neutral words significantly differed from each other; there was no significant difference between the arousal of positive words and negative words, while they both were significantly different from the neutral words. The familiarity and the number of strokes of characters were matched between conditions.

**Table 1:** Means (SDs) of the measured indices of the key words and sentential contexts.

**Statistic**	**Concrete word**			**Abstract word**		
	**Negative**	**Neutral**	**Positive**	**Negative**	**Neutral**	**Positive**
Valence	2.9 (0.69)	5.0 (0.28)	6.6 (0.54)	2.9 (0.44)	5.0 (0.34)	6.6 (0.44)
Arousal	5.8 (0.60)	4.5 (0.61)	6.1 (0.57)	5.9 (0.50)	4.2 (0.51)	5.9 (0.50)
Concreteness	7.8 (0.24)	7.8 (0.36)	7.7 (0.36)	3.5 (0.59)	3.7 (0.62)	3.8 (0.54)
Familiarity	6.7 (0.55)	6.7 (0.39)	6.5 (0.37)	6.5 (0.50)	6.7 (0.32)	6.6 (0.54)
Stroke of the real words	16.9 (4.62)	17.7 (4.73)	16.9 (5.33)	17.2 (4.22)	16.9 (3.71)	18.5 (3.73)
Stroke of the novel words	17.5 (4.10)	18.1 (4.23)	17.6 (4.62)	17.7 (3.52)	17.2 (3.43)	17.9 (4.34)
Sentences' valence	2.62 (0.57)	5.14 (0.28)	6.71 (0.71)	2.49 (0.48)	5.09 (0.36)	6.76 (0.72)
Sentences' arousal	5.79 (0.58)	4.56 (0.52)	5.86 (0.55)	5.84 (0.48)	4.16 (0.51)	5.87 (0.52)
Cloze probability	0.96 (0.20)	0.94 (0.24)	0.97 (0.18)	0.95 (0.21)	0.96 (0.22)	0.95 (0.21)

A total of 180 novel meaningless words were constructed of two random Chinese characters and were paired with the selected 180 real words to make up the key word pairs (e.g., novel word “扔里/rengli” vs. real word “足球/soccer”). Later, we compiled 180 Chinese sentential contexts that were consistent with the semantic and emotional information of the keywords (e.g., neutral/concrete: Brazilians like to play *rengli/soccer*).The examples for each condition are shown in Appendix A. Except for the keyword, each sentence background included 8–11 characters. Cloze probability, which is the proportion of people who complete a particular sentence fragment with that particular word (Taylor, 1953), was assessed by presenting each sentence to 20 new participants; the average correct rate was over 90% (see Table 1). We also evaluated valence and arousal of sentential contexts, and the values were consistent with valence and arousal of the key words (see Table 1).

#### Procedure

**Figure 1:**
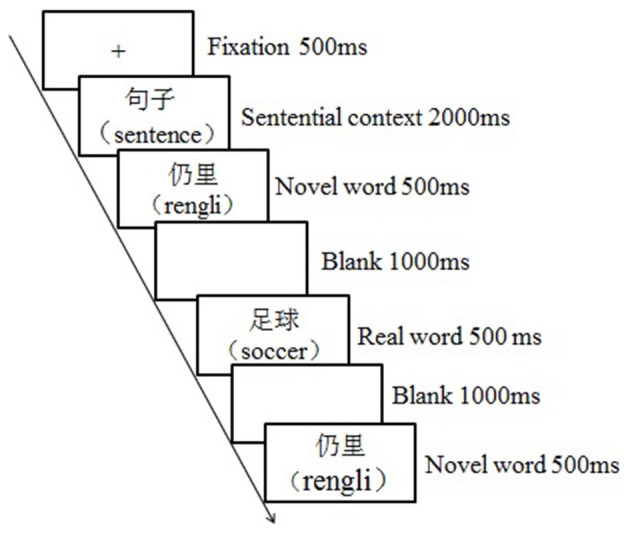
The illustration of learning trial.

**Figure 2:**
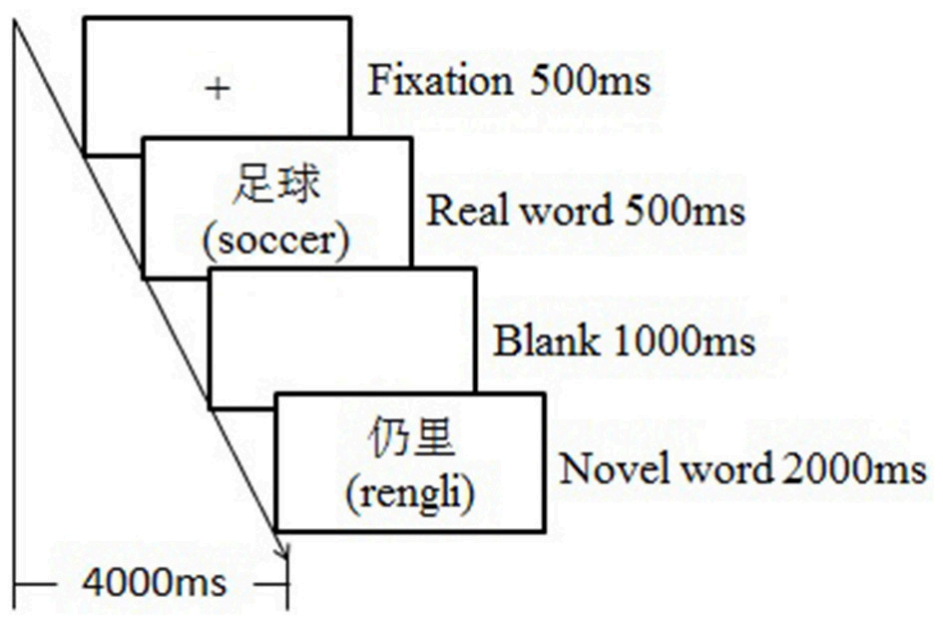
The illustration of testing trial.

The whole experiment lasted for 3 days for each participant. Each day, there was one behavioral training session followed by a testing session in the fMRI scanner. The training session lasted about an hour with each word learned three times, and the testing session followed the training session after a delay of around 10 min. During the training session, participants were asked to guess and remember the target novel words based on the emotional or neutral contexts. Specifically, a learning trial began with a fixation “+” for 500 ms, followed by an emotional or neutral context for 2,000 ms, then the key novel word appeared for 500 ms, and the participants were required to guess the meaning of it. After a 1,000-ms blank screen, the correspondent real word appeared for 500 ms, and then the novel word appeared again for 500 ms after another 1,000-ms blank screen to facilitate the consolidation process (see Figure 1). During the testing session, participants were asked to judge if the novel words were semantically consistent or inconsistent with the previously presented real words by responding to the keypad compatible with the scanner. To be specific, a testing trial began with a fixation (500 ms), which was followed by a real word (500 ms), and then a novel word appeared (2,000 ms) (see Figure 2). Participants had to respond during this interval by pressing the left or right key, which was counterbalanced across participants. The testing session was separated into three runs; each run contained 60 trials, with an equal number of trials among conditions. Event related design was adopted, testing trials took up about 50% of the time in a run, and the rest of the time was “+” as null stimuli. The presenting sequence of trials and null stimuli were pseudo-randomized to obtain a high ratio of signal to noise. Each run began with the null stimuli for about 10 s, and it lasted for about 8.3 min.

Before the fMRI scanning, participants were first familiarized with the experimental procedure by 20 trials that did not appear in the formal experiment and subsequently performed the experiment in the scanner. The participant's head was secured by foam rubber to minimize movement.

#### Apparatus

The fMRI experiment was performed on a 3.0 T Siemens Sonata whole-body MRI scanner located in the Beijing Normal University. Functional scans were obtained by using a single-shot T2^*^-weighted gradient-echo planar imaging (EPI) sequence. The following scan parameters were used: TR/TE = 2,000 ms/30 ms, matrix = 64 × 64 voxels, FOV = 24 cm, flip angle = 90°, and slice thickness/gap = 4 mm/0.6 mm; 32 contiguous sagittal slices were acquired to cover the whole brain at 250 time points in each run. A high-resolution structural scan was obtained using a 3D T1-weighted pulse sequence (TR = 16 ms, TE = 5.2 ms, flip angle = 25°, 128 slices, matrix = 256 × 256 mm, voxel size = 1 × 1 × 1 mm, FOV = 26 cm).

#### fMRI data analysis

We used SPM8 (Wellcome Department of Cognitive Neurology, London, UK) implemented in Matlab for image preprocessing and statistical analyses. Standard preprocessing was conducted including slice timing correction, realignment, spatial normalization, and smoothing. The functional images were normalized to the EPI template with 2 × 2 × 2 mm^3^ spatial resolution and then smoothed with a Gaussian filter at full width at half maximum (FWHM) of 8 mm.

A general linear model was used to estimate the condition effects with the canonical hemodynamic response function (HRF) for each subject. The data were globally scaled and high-pass filtered at 128 s. Significant changes in the hemodynamic response for each participant and condition were assessed using a factorial design. Individual activation maps of the concrete and affective effects were estimated through t-contrasts: concrete—abstract; abstract—concrete; negative—neutral; positive—neutral. Individual images for the respective contrasts of interest were generated for each participant and were then subjected to second-level (group analyses) random effects analyses using a within-subject ANOVA model. For group analyses, threshold of *p* < 0.01 with FDR correction was applied.

Region of interest (ROI) analyses were conducted to clarify the modulation of contextual emotion on the brain functional plasticity induced by word learning. The regions reported in literature involved in semantic and affective processing were selected as ROIs, including the bilateral IFG, MTG, AG, FG, insula, anterior cingulate cortex (ACC), and amygdala. The overall emotional effect on the third day was computed with inclusive anatomical masks of the above ROIs defined by the automated anatomical labeling (AAL) template available in xjView (http://www.alivelearn.net/xjview/). The coordinates of the peak activation for the resulting ROIs were as follows: L IFG (−42, 12, 27), R IFG (33, 27, 15), L MTG (−51, −48, −3), R MTG (51, −39, 3), L Angular (−30, −51, 39), R Angular (30, −54, 39), L Insula (−30, 21, 3), R Insula (33, 21, 0), L FG (−42, −84, −15), R FG (42, −60, −24), L ACC (15, 24, 30), R ACC (15, 24, 30). A sphere with an 8-mm radius was drawn, centered on the peak activation voxel in SPM8 using the MarsBar toolbox (http://marsbar.sourceforge.net/). The amygdala is an important region in emotional processing; we used AAL in xjView to structurally define the amygdala ROI. Small volume correction (SVC, *p* < 0.01, false discovery rate corrected) was carried out for these a priori regions of interest (Worsley et al., 1996). We then extracted the percent signal changes of the peak voxels in the ROI regions that survived after small volume correction (SVC) by the MarsBar toolbox.

### Results

#### Behavioral results

The mean accuracy rate was 73.5%, which was much higher than the chance level, indicating that the participants responded to the stimuli in the expected manner. In addition, we only analyzed the correct trials and excluded the trials with more than ±3 standard deviation on reaction times (RTs). The accuracy and RTs are shown in Tables 2, 3.

**Table 2:** The means (SDs) of accuracy for different conditions.

	**Negative context**		**Neutral context**		**Positive context**	
	**Day 1**	**Day 3**	**Day 1**	**Day 3**	**Day 1**	**Day 3**
Abstract	0.59 (0.14)	0.79 (0.18)	0.64 (0.13)	0.84 (0.14)	0.61(0.13)	0.82 (0.17)
Concrete	0.65 (0.15)	0.86 (0.17)	0.67 (0.12)	0.87(0.15)	0.63 (0.13)	0.87 (0.17)

**Table 3:** The means (SDs) of RTs for different conditions.

	**Negative**		**Neutral**		**Positive**	
	**Day 1**	**Day 3**	**Day 1**	**Day 3**	**Day 1**	**Day 3**
Abstract	1015.48 (196.10)	850.69 (153.48)	996.04 (194.10)	794.01 (136.00)	1028.39 (224.79)	809.84 (142.51)
Concrete	1004.13 (199.77)	778.46 (134.28)	992.97 (226.69)	752.50 (124.15)	958.12 (159.83)	744.64 (129.55)

A 2 (Training Day: day 1/day 3) ^*^ 3(Contextual Emotion: negative/neutral/positive) ^*^ 2(Concreteness: concrete/abstract) repeated ANOVA was run on accuracy and RTs. The analysis on accuracy showed a significant main effect of training day [*M_*day 1 = 0.63 ± 0.03, *M_*day 3 = 0.84 ± 0.04, *F*_(1, 15)_ = 53.70, *p* < 0.001, η^2^ = 0.78] and concreteness [*M_*abstract = 0.71 ± 0.03, *M_*concrete = 0.76 ± 0.03, *F*_(1, 15)_ = 14.47, *p* = 0.002, η^2^ = 0.49]. The analysis on RTs indicated a significant main effect of training day [*M_*day 1 = 999.19 ± 46.53, *M*_day 3 = 788.36 ± 31.20, *F*_(1, 15)_ = 22.89, *p* < 0.001, η^2^ = 0.60] and concreteness [*M_*abstract = 915.74 ± 35.04, *M*_concrete = 871.80 ± 31.64, *F*_(1, 15)_ = 15.58, *p* = 0.001, η^2^ = 0.51]. Moreover, the main effect of contextual emotion also reached significance [*F*_(2, 30)_ = 3.17, *p* < 0.05, η^2^ = 0.18], and *post-hoc* analysis showed that the novel words learned in a negative context (921.19 ± 36.28) were processed slower than the words learned in neutral (883.88 ± 32.96) and positive contexts (885.25 ± 31.77).

Taken together, the behavioral results suggest that novel word proficiency increased through the learning process, and the final attainment for concrete words was higher than abstract words. More importantly, we found a significant contextual emotion effect, which suggested that negative emotion hindered novel word learning.

#### fMRI results

The BOLD activations for the effects of concreteness and contextual emotion are shown in Table 4 (day 1) and Table 5 (day 3).

**Table 4:** Regions showing concreteness, emotion main effects on first day.

		**Coordinate**				
**Region**	**BA**	**x**	**y**	**z**	**t**	**Cluster**
Concreteness (Concrete > Abstract)	No significant activations					
Concreteness (Abstract > Concrete)	No significant activations					
Emotion (Positive > Neutral)	No significant activations					
Emotion (Negative > Neutral)						
R Insula		27	36	9	5.24	48
L Tri inferior frontal gyrus	4/6	−42	24	15	6.03	85
L Precentral gyrus	6/9	−30	−9	42	6.67	309
L Inferior parietal gyrus	7	−27	−51	39	5.08	61
L Supplementary motor area	6	0	3	60	7.85	368

*Coordinates indicate location in Montreal Neurological Institude (MNI) space of the maximally significant voxel. Height threshold, P (FDR corrected) < 0.01, spatial extent threshold, 20 voxels. L, left hemisphere; R, right hemisphere; BA, Brodmann's area*.

**Table 5:** Regions showing concreteness, emotion main effects on third day.

**Region**		**Coordinate**				
	**BA**	**x**	**y**	**z**	**t**	**Cluster**
Concreteness (Concrete > Abstract)	No significant activations					
Concreteness (Abstract > Concrete)	No significant activations					
Emotion (Positive > Neutral)						
R Oper Inferior frontal gyrus	9	33	12	30	5.73	59
L Inferior temporal gyrus	37	−48	−57	−18	8.23	50
R Angular	40	33	−54	39	5.28	45
R Insula	13	33	21	−3	5.44	81
L Fusiform gyrus	7	−30	−60	48	5.87	78
Emotion (Negative > Neutral)						
L Insula	13	−24	24	3	8.83	107

*Coordinates indicate location in Montreal Neurological Institude (MNI) space of the maximally significant voxel. Height threshold, P (FDR corrected) < 0.01; spatial extent threshold, 20 voxels. L, left hemisphere; R, right hemisphere; BA, Brodmann's area*.

The negative context had a greater activation compared with the neutral context in the right insula, left tri IFG, left precuneus, left inferior parietal gyrus (IPG), and left supplementary motor area (SMA) on the first day; the positive context has no significant effect (see Table 4). The negative context induced greater activation than the neutral context in the left insula and the left SMA; the positive context had greater activity than the neutral context in the right oper IFG, left inferior temporal gyrus (ITG), right angular, right insula, and left FG on the third day (see Table 5).

**Figure 3:**
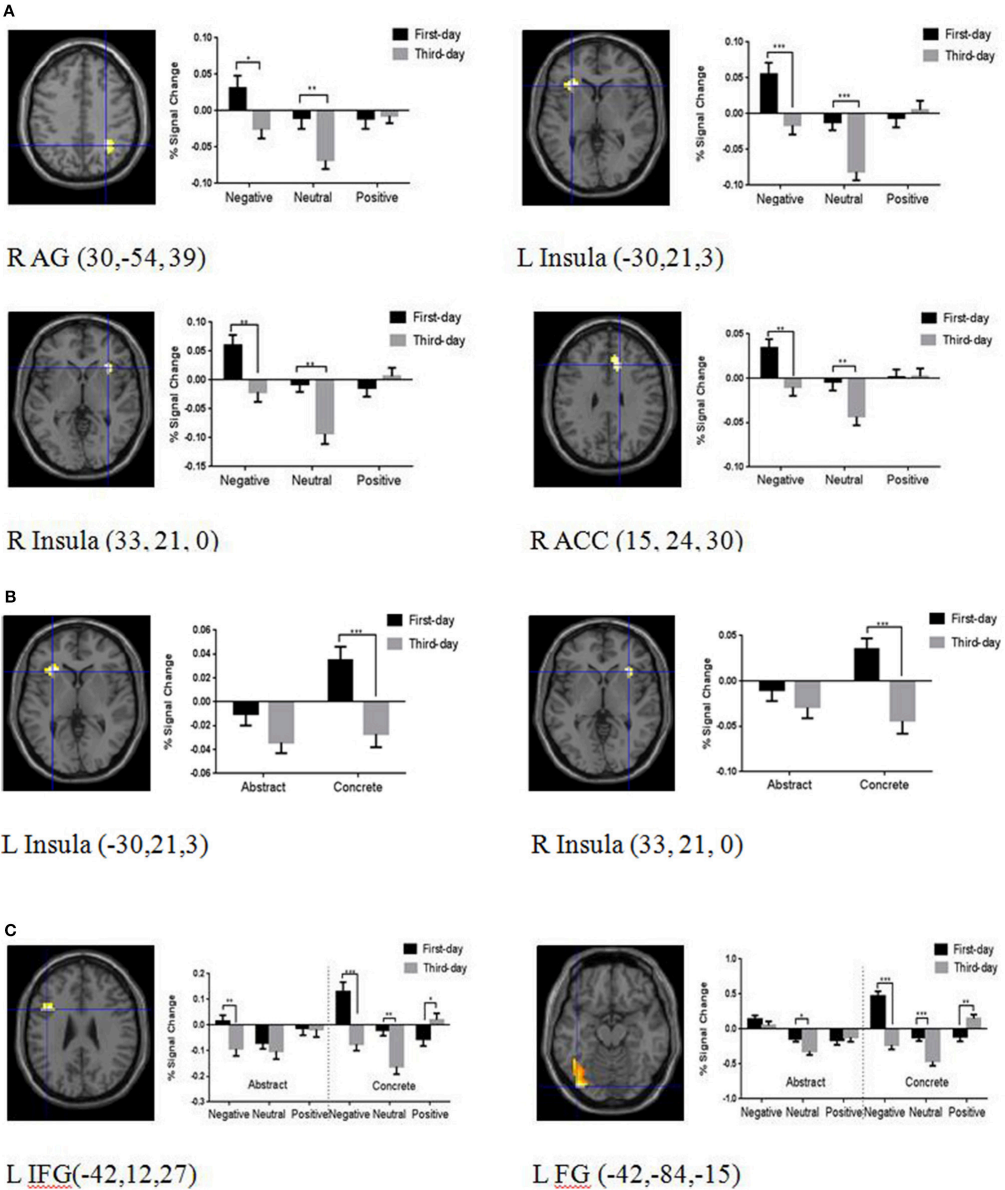
Brain regions and the mean percentage BOLD signal change illustrating the semantic and emotional processing areas. **(A)** Brain regions showing adaptive changes modulated by contextual emotion, including the right angular (AG), bilateral insula, and right anterior cingulate cortex (ACC). **(B)** Brain regions showing adaptive changes modulated by concreteness, including the bilateral insula. **(C)** Brain regions showing adaptive changes interactively modulated by contextual emotion and concreteness, including the left inferior frontal gyrus (IFG) and the left fusiform (FG). Results are superimposed on the T1 template image after applying a height threshold of *P* < 0.01, small volume corrected (SVC). The x, y, and z coordinates are in the MNI space. The error bars show standard errors. L, left hemisphere; R, right hemisphere. *P < 0.05, **P < 0.01, ***P < 0.001.

Six ROIs survived the *p* < 0.01 (SVC) threshold, including the left IFG, right AG, left FG, right ACC, left insula, and right insula. No significant activation was found in the structurally defined amygdala at *p* < 0.01 SVC. To explore the contextual emotion, concreteness, and their interactive effects on brain plasticity of word learning for the above six ROIs, 3 (Contextual Emotion) ^*^ 2 (Concreteness) ^*^ 2 (Training Day) within-subject ANOVAs were run on the percent signal change. There were significant interactions between training day and contextual emotion in the right AG, *F*_(2, 94)_ = 4.06, *p* < 0.05, η^2^ = 0.08; left insula, *F*_(2, 94)_ = 7.30, *p* = 0.001, η^2^ = 0.13; right insula, *F*_(2, 94)_ = 7.70, *p* = 0.001, η^2^ = 0.14; and right ACC, *F*_(2, 94)_ = 3.06, *p* = 0.05, η^2^ = 0.08. Simple effect analyses showed that the brain activations on day 3 were significantly reduced compared with day 1 only for the words learned in the negative and neutral contexts (*ps* < 0.01) (see Figure 3A).

There were also significant interactions between concreteness and training day in the left insula, *F*_(1, 47)_ = 4.33, *p* < 0.05, η^2^ = 0.08, and right insula, *F*_(1, 47)_ = 8.25, *p* = 0.001, η^2^ = 0.15. Simple effect analyses showed that the brain activations on the third day were adaptively reduced more than the first day only for concrete words (*p*s < 0.001) (see Figure 3B).

There were significant interactions between the three factors in the left IFG, *F*_(2, 94)_ = 3.18, *p* < 0.05, η^2^ = 0.06, and left FG, *F*_(2, 94)_ = 8.67, *p* = 0.001, η^2^ = 0.16. Simple effect analyses showed that the brain activations on day 1 were greater than day 3 for the concrete words learned in the negative and neutral contexts in the left IFG and FG (*p*s < 0.001), but the result pattern was reversed for the words learned in the positive context, where the activation was increased in day 3 rather than day 1 (*p*s < 0.01). There were no apparent emotional effects for abstract words except for the reduced brain activation of the left IFG on day 3 compared with day 1 in the negative context (*p* < 0.01) (see Figure 3C).

## Discussion

### Effects of contextual emotion and concreteness on learning behavior

The behavioral results revealed that the negative context hindered novel word learning compared with the neutral and positive contexts, which corresponds with the previous studies (Brand et al., 2007; Mao et al., 2015; Guo et al., 2018). Some researchers argued that emotions were informative: positive emotions carry “valuable,” “good,” and “easy” information while negative emotions carry “worthless,” “bad,” and “difficult” information (Chee et al., 2001; Bleser et al., 2003). The negative contextual information might reduce the relevance of the learned materials, so there was a lower attainment level of words learned in the negative emotional context. Additionally, the negative information from the contexts may catch more attention, therefore, novel word learning was impeded because of the lack of attention resources (Shafer et al., 2012). In a recent study, Guo et al. (2018) found that the negative emotional state delayed the word learning process especially at the beginning phase of learning. These results agreed on the fact that negative emotion from the learning context or learners' state could pose a significant impact on the novel word learning process.

The results also showed that learning concrete words had an advantage over abstract words, which was consistent with previous studies (Holcomb et al., 1999; West and Holcomb, 2000; Fliessbach et al., 2006; Mestres-Missé et al., 2014). In many behavioral studies, it has been demonstrated that concrete words have a processing advantage over abstract words (concreteness effect). Typically, abstract words are processed more slowly (Schwanenflugel and Shoben, 1983; Kroll and Merves, 1986) and take longer to read (Schwanenflugel and Shoben, 1983; Schwanenflugel et al., 1988) than concrete words. According to Paivio (1991), concrete words are presented both in verbal and image-based systems, which endow them with greater imageability to be more easily recalled compared with abstract words. Moreover, concrete words can also have more accessible semantic networks than abstract words (Schwanenflugel et al., 1992) so that they might be more easily put into contexts. When it comes to learning concrete words, learners can use vivid imagery information to facilitate the encoding process, so it is reasonable to find the enhancing effect for the learning of concrete words in our experiment.

### Modulation of contextual emotions on brain adaptive changes

Decreased activations on the third day relative to the first day were found across the right AG, bilateral insula, and right anterior cingulate gyrus in the negative context but not in the positive context. Such a decrease in activation indicated an evidence for a word learning effect, which could be confirmed by the better behavioral performance at day 3 than day1, and the learning effect was differently affected by negative and positive contextual emotions. Numerous studies have demonstrated that unpleasant or threatening stimuli take priority in cognitive processing (Charash and Mckay, 2002; Connolly et al., 2015; Macatee et al., 2017). Therefore, the participants may encounter more aversive emotional arousal on day 1 in the negative context compared with positive contextual emotion. With the progress of word learning, the participants began to adapt to the negative emotional information, leading to a reduction in the activation of the brain regions related to affective or cognitive processing. Some studies also suggested that sentential contexts enhance memory processing and additional emotion-specific processing (Maratos and Rugg, 2001; Maratos et al., 2001). Our results added to this body of evidence that negative information could be processed preferentially and interfere with people's attention, resulting in more cognitive resources for word learning.

Numerous studies have suggested that the AG is implicated in semantic processing (Dalgleish, 2004; Binder and Desai, 2011; Broek et al., 2016; Lesage et al., 2016). Molinaro et al. (2015) examined the neural dynamics underlying the semantic processing of different conceptual constructions and found dynamic connections between the anterior temporal cortex and AG for efficient semantic processing. The reduced activation in this region might suggest a greater efficiency of semantic processing of the novel target words, or we could infer that participants had acquired the semantic meaning of the novel words. Our results also provided effective support for the argument that the AG was also affected by contextual emotional information (Dolcos and Denkova, 2014), which was echoed by Smith et al. (2004) in finding that the left AG showed a significant emotional effect during both encoding and retrieval phases of word learning.

There was a debate on the role of the insula in conceptual and affective processing. Some researchers argued that the insula was a purely linguistic area involved in conceptual processing (Worsley et al., 1996; Rossell et al., 2001; Wu et al., 2012; Lesage et al., 2016), while others suggested the insula was an area responsible for affective processing (Liu et al., 2010; Zaki et al., 2012; Wager et al., 2013). Numerous studies have found that the insula is most commonly associated with the processing of disgust (Liu et al., 2015) and has also been reported in studies of fear conditioning (Merz et al., 2010) and instructed fear learning (Phelps et al., 2001). Our results revealed that the activation patterns of the insula were modulated by contextual emotions. This finding indicates that the insula is sensitive to the processing of contextual emotions, which suggests that this region is related to affective processing in accordance with the studies mentioned above. The insula was also linked to perceptual load and task difficulty in perceptual decision-making (Lamichhane et al., 2016), which made it seem like more insular activation was observed in a cognitively demanding task compared with an easy one. Such an explanation might be applicative to our results that lower insular activity was induced on the third day (i.e., easier task) compared with the first day.

The ACC is known to be involved in a form of attention that served to regulate both cognitive and emotional processing (Bush et al., 2000). Our results found that this region was involved in the word learning process and sensitive to the modulation of contextual emotion. Medford et al. (2005) examined whether the same neural processes underlay memory enhancement for both emotional target words and neutral target words encoded in an emotive context and found that the ACC was strongly activated by reading emotional sentences and recognizing emotional target words. They also found that ACC activation was negatively correlated with emotional target recognition, which provided direct evidence that this region modulated the impact of emotionally salient material, thus, influencing how it was encoded and retrieved. In addition, the ACC is considered to play an important role in response competition, as it triggers the engagement of cognitive control. Significant activation in the ACC has been reported in tasks that generate high attentional demands (Barch et al., 2000; Milham and Banich, 2005). So, activation in this region revealed that the negative contextual information might capture more attention and take up more cognitive resources.

### Modulation of words' concreteness on brain adaptive changes

We found that concrete words induced significant reduced activation in bilateral insula on the third day relative to the first day but abstract words did not. Concreteness was an important attribute of the word itself, and it is agreed upon that concrete words were easily processed and learned than abstract words. Concrete words showed more significant adaptive changes in the bilateral insula than abstract words, which suggested that the insula may be a key region sensitive to the conceptual attained level in collaboration with the behavioral results (Lesage et al., 2016). Some investigations have noted the contribution of the insula to conceptual processing. For example, a meta-analysis of fMRI studies on Chinese orthographic, phonological, and semantic processing showed that the group of orthographic and phonological processing induces a greater recruitment in the left insula as compared with semantic processing (Wu et al., 2012). Notably, the bilateral insula was also found to be associated with emotional modulation on learning effect in this study. Therefore, the insula may be a multifunctional region, which is shaped by learning experience.

### Emotional effects on brain plasticity modulated by concreteness of words

The decreased activation on the third day relative to the first day in the left IFG was found for both concrete and abstract words learned in the negative context but only for concrete words in the left FG. Our study also found the increased activation in the left IFG and FG for concrete words learned in the positive context but not for abstract words. These results showed that the IFG and FG were two important brain regions involving semantic and emotional processing modulated by word concreteness.

The IFG is an important brain region for semantic and lexical processing (Molinaro et al., 2015; Zhuang et al., 2016). Minicucci et al. (2013) used an auditory semantic priming lexical decision task and found that their results were consistent with the models of lexical-semantic access, identifying the role of the MTG in representing lexical-semantic information and the IFG in selecting amongst competing lexical items. Besides, some studies have also shown that the left frontal regions including the IFG were sensitive to sentential contexts, especially when speech intelligibility was low (Obleser et al., 2007; Obleser and Kotz, 2010), possibly because of the involvement of this region in semantic and contextual computation (Binder and Desai, 2011). Molinaro et al. (2015) also found that four out of five patients (80%) with lesions mostly in the left IFG showed impaired sentence processing, suggesting that this brain region played a critical role in sentence processing. Moreover, a number of studies found that the IFG was also related to affective processing. For instance, Kirby and Robinson (2014) found that five out of the seven emotions induced showed consistent activations within the amygdala, whereas all the emotions consistently activated the right IFG, which is considered as an integration hub for affective and cognitive processes. Roxbury et al. (2014) also revealed that the left IFG was consistently activated for anger, disgust, and fear. The interactive actions of contextual emotion and word concreteness in the IFG add to the evidence that the IFG is a key region for integrating semantic and emotional information.

The FG is an area implicated in orthographic processing (Binder et al., 2006) and visual word processing (Dehaene and Cohen, 2011; Twomey et al., 2011). In addition, the FG also has been shown to be sensitive to emotional manipulation. Many studies demonstrated that activation in the FG was modulated by the emotional valence of materials. Activations in the bilateral FG were found during viewing (Lane et al., 1997) and evaluating unpleasant pictures (Paradiso et al., 1999). A recent study conducted a meta-analysis on affective mapping and found that the left FG was associated with fear, anger, and disgust (Roxbury et al., 2014). Moreover, our results showed that abstract words did not show significant functional plasticity changes as compared with concrete words in the negative context, suggesting that the activation of the left FG was modulated by word concreteness (Fliessbach et al., 2006; Mestres-Missé et al., 2014). In the study of Fliessbach et al. (2006), there was a positive correlation between brain activity in the left anterior FG and the strength of the concreteness effect during recognition. Our study showed that the activity changes in the FG were bias observed for concrete words, which might be interpreted in accordance with the dual-coding theory. Concrete words have more vivid imagery than abstract words, and the fusiform activity was constantly found in visual processing (Fiebach and Friederici, 2004) and associated with the retrieval of visual object information (Price et al., 2003; Wheeler and Buckner, 2003), thus, leading to this finding of FG for concrete words rather than for abstract words.

Contrary to the negative context, we found the increased activation in the left IFG and FG in the positive context only for concrete words, which might suggest that the proficiency for the learned word reached a high level since positive context could facilitate the word learning process. In fact, the IFG has been found to be related to learning difficulty. Prat et al. (2012) conducted a volumetric analysis of activation in the right IFG showing linear increases in activation with difficulty. Raboyeau et al. (2010) also found that the early learning phase (low proficiency) was characterized by activations in the left IFG and Broca's area, which were associated with effortful lexical retrieval and phonological processing, respectively. Concrete words are constantly found to be easier to learn and can be attained at a higher proficiency. Therefore, our finding that concrete words learned in the positive context increased activations in the IFG is consistent with the previous studies (Wise et al., 2000; Whatmough et al., 2004; Sabsevitz et al., 2005; Wallentin et al., 2005; Bedny and Thompson-Schill, 2006; Fliessbach et al., 2006). Mestres-Missé et al. (2009) asked participants to read pairs of sentences in order to derive the meaning of new words that appeared in the terminal positions of the sentences and found that concrete word learning selectively boosted the activation in the ventral anterior FG, suggesting that the left FG was an important brain region related to word concreteness. The results supported our hypothesis in that the contextual emotions exerted different impacts on learning of concrete and abstract words, respectively.

The lack of activation of the amygdala in the emotional context was inconsistent with the previous research (Maratos and Rugg, 2001). A possibility was that the activation of the amygdala might be relevant to the task difficulty. Our study adopted a semantic judgment task, which might be more difficult than the above experiment tasks, such as a recognition memory test that only needs participants to discriminate between learned and unlearned words.

## Conclusion

In summary, we investigated the role of contextual emotions in word learning, employing a novel context-based word learning paradigm. This study identified the cerebral substrates for the influence of contextual emotions on different types of words. The right AG, left/right insula, and anteior cingulate gyrus are important brain regions that showed functional plasticity changes modulated by the contextual emotion. Besides, our study helped to clarify the role of the insula, which is involved in both conceptual and affective processing. More importantly, the adaptive changes in the left IFG and FG induced by word learning were differently modulated by contextual emotion for concrete and abstract words. These results suggest that situational contexts could pose remarkable impact on word learning and its related brain plasticity changes even for adult learners. Language teachers should pay more attention to the display of emotional context during the learning of new words especially for concrete words. However, it would be helpful to investigate foreign language learning in a more authentic learning environment in the future. In addition, the results showed interhemispheric differences for contextual emotion modulation, which needs more specific studies to explore these potential findings.

## Ethics statement

This study was carried out in accordance with the recommendations of the Institutional Review Board of BNU. The protocol was approved by the Institutional Review Board of BNU. All subjects gave written informed consent.

## Author contributions

JG and CC conceptualized this study. JG and DL contributed to the implementation of data collection and analysis. JG, YB, and DL drafted the manuscript.

### Conflict of interest statement

The authors declare that the research was conducted in the absence of any commercial or financial relationships that could be construed as a potential conflict of interest.

## Appendix A

Examples of experimental conditions.

**Table:**

Negative and concrete	She was sentenced to five years and lived in a woman ____ 她被判五年,住进了女子____	Jiangzhou/浆舟	Prison/监狱
Negative and abstract	The boss for his staff is constantly putting ____ 老板不断对她施加____	Xizhang/习张	Pressure/压力
Neutral and concrete	Brazilians like to play ____ 巴西人喜欢踢____	Rengli/仍里	Soccer/足球
Neutral and abstract	Calcium, iron, and sodium are all chemical ____ 钙，铁，钠都是化学____	Zhuanglin/庄林	Element/元素
Positive and concrete	After blowing out the candles, they began to eat the birthday____ 吹灭生日蜡烛,她们开始___	Jiaolu/觉陆	Cake/蛋糕
Positive and abstract	She received an unexpected gift, full of___ 她收到意外的物，感到很___	Ladai/蜡代	Surprise/惊喜___
